# Active and highly durable supported catalysts for proton exchange membrane electrolysers†

**DOI:** 10.1039/d4ey00026a

**Published:** 2024-06-21

**Authors:** Debora Belami, Matthew Lindley, Umesh S. Jonnalagadda, Annie Mae Goncalves Bullock, Qianwenhao Fan, Wen Liu, Sarah J. Haigh, James Kwan, Yagya N. Regmi, Laurie A. King

**Affiliations:** a Faculty of Science and Engineering, Manchester Metropolitan University Chester Street M1 5GD UK y.regmi@mmu.ac.uk l.king@mmu.ac.uk; b Department of Materials, University of Manchester Oxford Road Manchester M13 9PL UK; c Department of Engineering Science, University of Oxford Parks Road Oxford OX1 3PJ UK; d School of Chemistry, Chemical Engineering and Biotechnology, Nanyang Technological University 62 Nanyang Drive 637459 Singapore

## Abstract

The design and development of supported catalysts for the oxygen evolution reaction (OER) is a promising pathway to reducing iridium loading in proton exchange membrane water electrolysers. However, supported catalysts often suffer from poor activity and durability, particularly when deployed in membrane electrode assemblies. In this work, we deploy iridium coated hollow titanium dioxide particles as OER catalysts to achieve higher Ir mass activities than the leading commercial catalysts. Critically, we demonstrate state-of-the-art durabilities for supported iridium catalysts when compared against the previously reported values for analogous device architectures, operating conditions and accelerated stress test profiles. Through extensive materials characterisations alongside rotating disk electrode measurements, we investigate the role of conductivity, morphology, oxidation state and crystallinity on the OER electrochemical performance. Our work highlights a new supported catalyst design that unlocks high-performance OER activity and durability in commercially relevant testing configurations.

## Abstract 2

Hollow titanium dioxide supported iridium oxygen evolution reaction catalysts exhibit high activity and durability for the half cell and membrane electrode assembly testing protocols.

## Graphical abstract

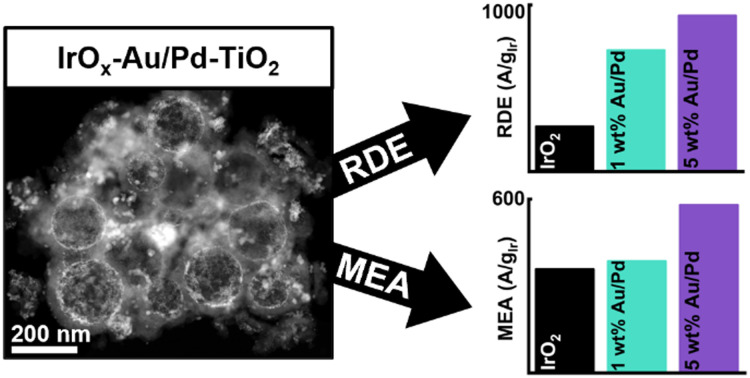

**Broader context**

Proton exchange membrane water electrolysers (PEM-ELs) are a promising technology that can produce low-cost green hydrogen. However, large scale commercialisation of PEM-ELs requires the use of high loadings (>2 mg cm^−2^) of expensive and scarce iridium to drive the oxygen evolution reaction. The ability to produce active and durable catalysts for long-term use without any Ir has long been a major challenge within this field. Many efforts have focused on reducing Ir (<75 wt%) by synthesising Ir-based supported catalysts. However, to date, such supported catalysts have shown insufficient stability under commercially relevant durability testing. To address this critical challenge, we report a unique water oxidation catalyst that deploys a Ir–AuPd–TiO_2_ OER catalysts which demonstrates excellent performance under acidic conditions. We assess the catalysts in both a half-cell rotating disk electrode setup as well as in a membrane electrode assembly. Impressively, after 10 000 accelerated stress test cycles, our catalyst has a comparable degradation rate to commercial Ir catalysts.

## 1. Introduction

The increasing environmental threats from rising global temperatures due to fossil fuel combustion has expedited the demand for clean renewable energy. However, the intermittent and fluctuating nature of renewable energy technologies such as wind and solar inherently requires coupling to energy storage technologies.^1^ Electrolytic hydrogen has been shown to be a sustainable energy carrier^2^ and can provide storage for renewable energy.^3^ Additionally, hydrogen is a feedstock in the chemical industry, as part of a mix of gases in steel production, fertiliser production, and can be used for heat and power generation. In 2021, the total annual production of hydrogen reached 94 million tonnes (Mt) globally.^4^ The majority (95%) of hydrogen is produced *via* hydrocarbon reforming which emits CO_2_.^5,6^ Indeed, the 90 Mt produced globally in 2020 emitted approximately 900 Mt CO_2_.^7^ The demand for hydrogen is expected to grow rapidly in the near future. Zero carbon methods of hydrogen production, such as electrolysis powered by renewable electricity, is a promising pathway to both store renewable energy and decarbonising such critical industrial chemical processes.^8^

Several types of electrolysers exist today including proton exchange membrane water electrolysers (PEM-ELs) which are considered promising owing to their high efficiency and production of high purity H_2_.^8^ However, current commercial PEM-ELs require the use of expensive and scarce Ir-based catalysts at relatively high loadings (>2 mg_Ir_ cm^−2^)^9,10^ to achieve optimum activity and durability, largely due to the sluggish oxygen evolution reaction (OER).^10,11^ The high cost and low abundance of Ir represent a significant barrier to the scalability of the technology.^12,13^ Therefore, the development of high performance and cost effective OER electrocatalysts is critical to the successful widespread deployment of PEM-ELs.^14^

There have been many efforts focused on reducing Ir loading without compromising electrochemical activity and stability. For example, highly active electrocatalysts have been sought by employing mixed metal oxides (*e.g.* perovskites or pyrochlores), or by modifying the electrocatalyst's morphology to generate core–shell nanoparticles,^15–17^ nanorods,^18,19^ or nanowires.^20^ The development of supported Ir catalysts represents an alternative, and highly desirable pathway towards high-performance catalysts at drastically lower Ir loadings.^21–24^ To be viable in electrochemical applications, catalyst supports themselves must also have high chemical stability, sufficient electrical conductivity, high surface areas and be cost-effective.^14,25^ Metal oxides have recently received significant attention as supports for Ir catalysts for PEM-ELs, however, metal oxides inherently demonstrate low conductivity and so to mitigate this doped metal oxides have been explored.^26,27^ In particular, antimony doped tin oxide has gained attention due to its high conductivity and enhanced Ir utilisation, achieving high OER activity and stability under acidic environments.^21,28,29^ However, studies have shown that in many cases the dopant (Sb) leaches over time, thereby reducing the stability of the support and thus inducing the dissolution of the Ir catalysts.^30,31^ Consequently, exploring alternative strategies to develop high performance catalyst supports remains a critical challenge to fully exploit the potential of PEM-ELs at scale.

Amongst metal oxides, titanium dioxide (TiO_2_) is a very attractive OER catalyst-support because it has high resistance to corrosion and several TiO_2_ synthesis routes have been developed to prepare high surface area nanostructures.^32^ Despite these attributes, the low conductivity of TiO_2_ is a hinderance, with Ir loadings as high as 75 wt% required to achieve commercially relevant OER activities.^33^ Strategies to increase the conductivity of TiO_2_ have therefore been explored, including the synthesis of sub-stochiometric oxides.^26,34,35^ Nanostructuring strategies such as core–shells^15,36–38^ and high surface area TiO_2_^33^ have also been employed to maximise Ir utilisation. Conductive layer coated supports (CCSs), where a conductive Pt interlayer is introduced between the TiO_2_ core and Ir catalyst, have recently shown 141% higher OER mass activity compared to commercial 75 wt% IrO_2_–TiO_2_ in a rotating disk electrode (RDE).^9^ The nanostructured conductive interlayer allows for a lower total platinum-group metal (PGM) loading and simultaneously enhanced conductivity and is thus a promising approach for lowering Ir catalyst loading in PEM-ELs.

In this study, we investigate a new catalyst-support morphology, deploying a hollow TiO_2_ support coated with alloyed AuPd nanoparticles (AuPd–TiO_2_). We evaluate the effects of AuPd nanoparticle loading (1 wt% and 5 wt%), Ir loading (25 wt% and 50 wt%) and annealing environment (reducing and oxidised) to decipher how these factors correlate with catalyst conductivity and OER activity. A previous study by Oakton *et al.* has shown that the conductivity precipitously decreases below 40 mol% Ir content.^12^ Thus, we selected a composition (25%) from low conductivity region and another from a region (50%) where conductivity starts to exponentially increase in this study. The electrochemical performance is assessed in both RDE half-cell setup and in a membrane electrode assembly (MEA) enabling comparisons between these two distinct electrochemical testing configurations. By RDE, we observe the highest mass activities (936 A g_Ir_^−1^ at 1.65 V_RHE_) for 50 wt% Ir supported on 5 wt% AuPd–TiO_2_ annealed in a reducing atmosphere (50-WH5-H_2_) outperforming the 1 wt% AuPd–TiO_2_ and uncoated TiO_2_ supports. Interestingly, the highest performing catalyst tested in RDE was also the highest performing catalysts in MEAs when subjected to 10k accelerated stress test (AST) potential cycles. Specifically, 50-WH5-H_2_ reached 1 A cm^−2^ at 1.82 V whereas the less conductive 50 wt% Ir–1 wt% AuPd–TiO_2_ (50-WH1-H_2_) required 1.88 V to reach 1 A cm^−2^ after 10k AST cycles. Furthermore, the average voltage decay for the highest performing catalyst was 3 μV per cycle at 1 A cm^−2^ which is comparable to state-of-the-art IrO_2_ catalysts.^39^ Such high durabilities for supported catalysts demonstrate that our catalysts are state-of-the-art when compared against reported materials in the literature for PEM-ELs under analogous MEA architectures, operating conditions and accelerated stress test profiles. Analysis using X-ray diffraction (XRD), X-ray photoelectron spectroscopy (XPS) and electron microscopy show that the Ir catalysts maintain the as-synthesised structural and electronic properties after AST in an MEA, which we hypothesise supports the material's high electrochemical stability.

## 2. Experimental section

### 2.1. Synthesis of hollow TiO_2_ and AuPd–TiO_2_

The synthesis of hollow TiO_2_ and AuPd–TiO_2_ nanostructures has been previously reported elsewhere.^40^ In brief, the TiO_2_ nanostructured supports were synthesised by a sol–gel templating method, whereby polystyrene beads were coated with TiOH gel and calcined at 500 °C. The TiO_2_ supports were subsequently decorated with AuPd alloy nanoparticles (Au/Pd w/w = 5 : 1). To integrate AuPd alloy nanoparticles, Au nanoparticles were initially synthesised by reducing HAuCl_4_·3H_2_O^41^ followed by alloying with Pd *via* a modified seed-mediated process.^42^ The AuPd nanoparticles were then added dropwise to a dispersion of the previously synthesised TiO_2_ while stirring at room temperature. After 2 hours, the powder was recovered by centrifugation and dried at 70 °C overnight. Two different nanoparticle loadings were prepared, which we label “WH1” and “WH5”. The AuPd loading was determined for each sample *via* ICP-OES (Table S1, ESI†) with WH1 the lower loading of 1.17 wt% Au and 0.26 wt% Pd and WH5 having the higher loading of 5.54 wt% Au and 1.08 wt% Pd.

### 2.2. Synthesis of Ir-WH1 and Ir-WH5

The deposition of iridium (Ir) onto the nanostructured TiO_2_ and AuPd–TiO_2_ supports (WH1 and WH5) was performed by a process adapted from a previously published incipient wetness impregnation synthesis.^36^ In brief, 134 mg of dihydrogen hexachloroiridate(iv) hydrate (H_2_IrCl_6_·6H_2_O, 40% Ir, Acros Organics) was mixed with 20 μL deionised H_2_O (18.2 MΩ, Milli-Q) and 40 μL acetic acid (99.7%, Fisher Scientific). Separately, each support (hollow TiO_2_, WH1 and WH5) was dispersed in 20 mL of ethanol to obtain a homogenous solution. The mass of the support was varied to enable control of Ir loading (25 wt% and 50 wt%). The prepared Ir and support dispersion were subsequently bath sonicated for 10 min, then stirred at 80–100 °C until dry.

Each dried supported catalyst powder was collected and finely ground using a pestle and mortar. Each powder was placed into a tube furnace, ramped to 500 °C at 10 °C min^−1^ and held for 2 hours at temperature under a reducing atmosphere (5% H_2_ and 95% N_2_) at 50 sccm. The furnace was then allowed to cool to room temperature and a controlled passivation was conducted by flowing O_2_ (50 sccm) for 20 s. The resulting powders were then finely ground again. Each of the 50 wt% Ir supported catalysts were split into two batches and one half was subsequently annealed further in a box furnace (500 °C at 10 °C min^−1^ under atmospheric conditions for 30 minutes), cooled to room temperature and ground again using a pestle and mortar. The other half of these samples, which had only been annealed under a reducing atmosphere were tested as prepared.

### 2.3. Physical characterisation

The crystal structure of the catalyst-supports was examined by XRD using PANalytical X’pert powder X-ray diffractometer with a Cu Kα source (*λ* = 1.5406 Å). Diffraction peaks were recorded in the range 2*θ* = 20°–60° with a step size of 0.013°, a measurement time of 89 seconds per step and a sample rotation rate of 60 rpm. The reference patterns were identified using the Inorganic Crystal Structure Database (ICSD) and Scherrer analysis was performed on the 2*θ* peaks at 25.0° and 40.8° for Ir and Ti, respectively. XPS was performed using a Kratos Axis Supra system with a monochromated Al Kα_1_ radiation (*hv* = 1486.6 eV). The survey spectra are collected with a pass energy of 160 eV and region scans with a pass energy of 40 eV. All spectra were energy shifted to align to the C 1s peak at 284.8 eV. The morphologies of the supports were determined on a Zeiss Supra 40VP scanning electron microscopy (SEM) with EDAX 40VP energy dispersive X-ray (EDX) analyser. High resolution transmission electron microscopy (HRTEM) imaging of the catalysts was performed using an FEI Talos F200X and scanning TEM (STEM) was acquired using a probe aberration corrected FEI Titan G2 80-200 ChemiSTEM, with both microscopes operated at 200 kV. Particle sizes were determined using high-angle annular dark field (HAADF) STEM imaging, collected using 110 pA beam current, 21 mrad convergence angle, and a 48 mrad HAADF inner collection angle. STEM elemental mapping was performed by EDX spectroscopy using a Super-X quad silicon drift detector with a total collection angle of 0.7 sr. TEM samples were prepared by dry powder deposition onto holey carbon support films on 400 mesh Cu grids (Agar Scientific). The statistical analysis of Au and Ti particle sizes were determined using GraphPad Prism version 9.5.1 for Windows (GraphPad Software, San Diego, California USA). The statistical normally distributed data were compared with an unpaired *t*-test and non-normally distributed data were compared with Mann–Whitney *U* tests. The categorical frequency data were compared using the chi-squared test with *post hoc* Fisher's exact testing and results were deemed significant if *p* < 0.05. The elemental dissolution of the catalyst-supports was determined by analysing aliquots of electrolyte taken post electrochemical testing using inductively coupled plasma mass spectrometry (ICP-MS, Agilent 7900). The metal loading of the supports was determined by inductively coupled plasma optical emission spectrometry (ICP-OES, Agilent 5800), where 10 mg of AuPd–TiO_2_ was digested in 10 mL of aqua regia, filtered and diluted to 50 mL.

### 2.4. Conductivity measurements

The conductivities of the catalyst powders were measured in a 2-electrode conductivity cell developed in house, analogous to those previously reported in the literature.^43–45^ In brief, two copper electrodes were wrapped in a hole punched PTFE (Polyfon, thickness of 0.127 mm) to isolate the two electrodes. The PTFE hole acted as a sample holder for powder samples. The powder samples were then compressed between the electrodes under constant pressure at 500 psi. The conductivity cell was connected to a potentiostat (Metrohm, PGSTAT 204) to generate *I*–*V* curves using linear sweep voltammetry (LSV) from −0.4 to 0.4 V, to determine the electrical resistance and the conductivity calculations are discussed in the ESI.†

### 2.5. Half-cell electrochemical characterisation

Half-cell electrochemical measurements were conducted on a RDE (Pine Research) and VSP-3e potentiostat from BioLogic Science Instruments in a three-electrode configuration. A 0.1 M HClO_4_ electrolyte (pH 1.1), gold disk working electrode (0.196 cm^2^), graphite rod counter electrode and Hg/HgSO_4_ reference electrode (OrigaSens, Alvatek) were used throughout. The synthesised supported catalysts were compared against commercial iridium(iv) oxide (IrO_2_, Premion 99.99%, Alfa Aesar). All potentials were adjusted to the reversible hydrogen electrode (RHE) as detailed in the ESI.†

Catalysts were deposited onto the gold disk working electrode using a previously reported modified method.^9,46^ The catalyst inks were prepared by dispersing 2 mg of catalyst powder in 360 μL 1-propanol (NPA, 99%, Fisher Scientific), 120 μL deionised water (18.2 MΩ, Milli-Q) and 20 μL Nafion™ (5 wt%, Alfa Aesar). Inks were bath sonicated (FB15048, Fisherbrand) for 30 min prior to electrochemical testing followed by immediately drop casting onto the RDE Au disk at 200 rpm. The disk was subsequently rotated at 700 rpm for at least 30 minutes until dry. The theoretical Ir loading of each catalyst was 25.5 μg_Ir_ cm^−2^. Each synthesised catalyst ink was deposited and tested 3 times. Between each deposition, the electrode was polished with 0.05 μm alumina slurry, rinsed and sonicated for 30 seconds in deionised water.

Prior to electrochemical OER testing, potentiostatic electrochemical impedance spectroscopy (PEIS) measurements with 85% correction were conducted at 1.1 V_RHE_ (100 mHz to 200 kHz) and used to iR correct the reported potentials. The catalyst deposited working electrode was conditioned using cyclic voltammetry (CV) between 0.025–1.0 V_RHE_ for 50 cycles at 200 mV s^−1^. The OER activity was subsequently assessed at 2500 rpm using CV by sweeping the potential between 1.0–1.8 V_RHE_ at 10 mV s^−1^ for 10 cycles. We used the performance of 10th CV cycle for analysis unless otherwise stated. To examine the stability in an RDE configuration, a CV was measured from 1.0–1.8 V_RHE_ for 30 cycles at 10 mV s^−1^ and 2500 rpm.

### 2.6. Membrane electrode assembly

Catalyst inks were prepared following a method described elsewhere.^47^ Briefly, commercial 50 wt% Pt/C, deionised water, NPA and Nafion™ were mixed and bath sonicated for 30 minutes. The supported catalyst ink for the anode was mixed with deionised water, NPA and Nafion™ and horn sonicated (505, Fisherbrand) for 30 minutes. The vial was covered with parafilm and housed in an ice bath throughout sonication.

The catalyst coated membranes (CCMs) were prepared by drop casting the catalyst inks onto PTFE films on a vacuum hot plate at 53 °C and left to dry. Decal transfer was achieved by sandwiching the Nafion membrane (N212, Chemours) between the catalyst deposited PTFE films using a hot press under 1 metric ton pressure at 130 °C for 3 minutes.^48^ The catalyst loading is determined *via* gravimetric measurements of the PTFE before and after the decal transfer. The anode catalyst loading ranged between 1.58–1.62 mg_Cat_ cm^−2^ and cathode catalyst loading was 0.7–0.84 mg_Cat_ cm^−2^.

The CCMs were integrated into a single cell electrolyser with Pt-plated single serpentine 5 cm^2^ titanium flow field on the anode, single serpentine 5 cm^2^ graphite flow field on the cathode, water feed at 120 mL min^−1^ on the anode and N_2_ (100% RH) at 50 sccm at the cathode. Platinised sintered titanium porous transport layers (PTLs) from Mott corporation USA were used on the anode and carbon cloth gas diffusion layers (GDLs) with mesoporous layer (MPL) from FuelCellStore on the cathode. The cell was equilibrated at 60 °C for an hour prior to collecting polarisation curves using a BioLogic potentiostat (VSP-3e) with a 20 A booster (VMP3B-20). PEM-EL polarisation curves were generated *via* chronoamperometry (CA) steps collected for 3 minutes at increments of 0.05 V from 1.2–2.0 V. For each voltage step, the average current density of the last 30 s was used for the polarisation curve. For the saw-tooth voltage cycling accelerated stress tests (ASTs), 10k CVs were collected at 50 mV s^−1^ between 1.20 and 2.00 V. Polarisation curves were collected after each 1k cycles to assess performance degradation due to AST CVs. The concentration of dissolved Ir from the supported catalysts post-AST were evaluated by conducting inductively coupled plasma mass spectrometry (ICP-MS) on a 5 mL aliquot of the anode water outlet at the end of the ASTs.

## 3. Results and discussions

### 3.1. Electrochemical half-cell performance

In this study, nanostructured hollow TiO_2_ catalyst supports were utilised as catalyst supports. The prepared nanostructures consisted of Ir deposited by wet impregnation onto AuPd–TiO_2_ supports (WH1 and WH5) and TiO_2_ supports (without AuPd). The catalyst powders were subjected to either: (1) a thermal reduction (5% H_2_/95% N_2_) (TiO_2_-H_2_, WH1-H_2_ and WH5-H_2_) or (2) a thermal reduction (5% H_2_/95% N_2_) followed by annealing in air (TiO_2_-Air, WH1-Air and WH5-Air). For each catalyst prepared, two different Ir loadings (25 wt% and 50 wt%) were prepared. The samples are labelled as follows, “Ir wt%-catalyst support-annealing environment” as summarised in Table 1 alongside key synthetic details.

**Table 1:** Summary of supported catalysts prepared including sample names and brief synthetic details. The weight fractions of the catalyst-supports (without Ir) are provided in Table S1 (ESI)

Sample name	Ir wt%	Support	Annealing environment	Annealing programme
50-TiO_2_-H_2_	50	Open nanoshell TiO_2_	5% H_2_/95% N_2_	500 °C, 2 hours, 10 °C min^−1^
50-WH1-H_2_	50	1.1% Au/0.3% Pd–TiO_2_	5% H_2_/95% N_2_	
50-WH5-H_2_	50	5.5% Au/1.2% Pd–TiO_2_	5% H_2_/95% N_2_	
25-WH1-H_2_	25	1.1% Au/0.3% Pd–TiO_2_	5% H_2_/95% N_2_	
25-WH5-H_2_	25	5.5% Au/1.2% Pd–TiO_2_	5% H_2_/95% N_2_	
50-TiO_2_-Air	50	TiO_2_	1. 5% H_2_/95% N_2_	1. 500 °C, 2 hours, 10 °C min^−1^
			2. Air	
50-WH1-Air	50	1.1% Au/0.3% Pd–TiO_2_	1. 5% H_2_/95% N_2_	
				2. 500 °C, 0.5 hours, 10 °C min^−1^
			2. Air	
50-WH5-Air	50	5.5% Au/1.2% Pd–TiO_2_	1. 5% H_2_/95% N_2_	
			2. Air	

**Fig. 1:**
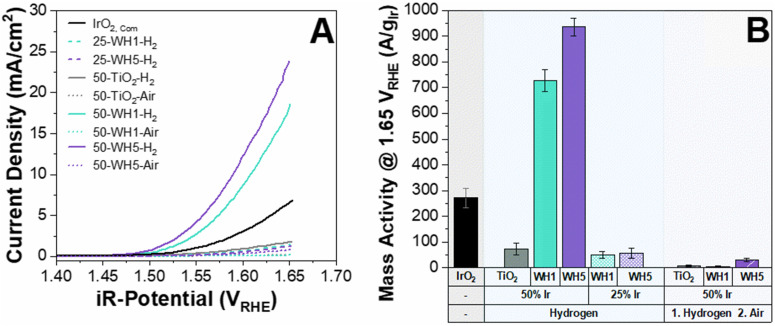
(A) Geometric area normalised OER activities of supported Ir catalysts and (B) OER activities normalised to the mass of Ir. All experiments were conducted at least in triplicates to generate error bars. The electrolyte was 0.1 M HClO_4_ electrolyte. Activities are reported for the 10th CV cycle with a theoretical Ir loading of 25.5 μg_Ir_ cm^−2^ and compared against unsupported commercial rutile IrO_2_ sample.

The activity of the prepared catalyst-supports were initially assessed using a three-electrode RDE in acidic electrolyte (0.1 M HClO_4_). Typically, 0.5 M H_2_SO_4_ is used for electrochemical measurements however, within this work 0.1 M HClO_4_ was utilised to conduct the electrochemical half-cell measurements to mitigate the strong binding effect of SO_4_^−^ to the catalyst which hinders the OER performance.^49^ It has been suggested that more electronegative anions have higher OER performance as it increases the likelihood of the ionic dissociation from the surface, thereby leaving more active sites exposed for OER.^50^ When catalysts are compared on a geometric current density basis, it is clear that the supported catalysts containing AuPd nanoparticles (WH1 and WH5) exhibited vastly superior OER performance over bare TiO_2_ (Fig. 1A). In addition to the presence of AuPd, thermal annealing conditions and iridium loading also had significant impacts on OER activities. Specifically, the average geometric current density at 1.65 V_RHE_ for 50 wt% Ir loaded supported catalysts annealed in H_2_ is 21.2 mA cm^−2^. When the same material is subsequently annealed in air, the current density is reduced to 0.5 mA cm^−2^ (Fig. 1A). This is in agreement with previous literature whereby the annealing environment (air) led to decreased activities.^51^ Two distinct Ir loadings were synthesised and annealed under a reducing environment: 25 wt% and 50 wt% for both WH1 and WH5 supports. The average geometric area normalised current densities at 1.65 V_RHE_ for 25 wt% Ir are 1.4 mA cm^−2^ and 21.2 mA cm^−2^ for 50 wt% Ir, respectively (Fig. 1A).

The electrochemical impedance spectroscopy was measured between 100 mHz–200 kHz prior to the OER measurements to determine the ohmic resistance of each supported catalyst (Fig. S1, ESI†). The fitting is an estimation based on a modified Randles circuit.^52,53^ We observed that the ohmic resistance was <30 ohm for AuPd–TiO_2_ based samples annealed under H_2_ conditions compared to >30 Ohm for similar samples under air annealing. Interestingly, the Ohmic resistance of 25 wt% Ir supported catalysts was similar to its 50 wt% Ir counterpart despite lower OER performance (Fig. 1B).

**Fig. 2:**
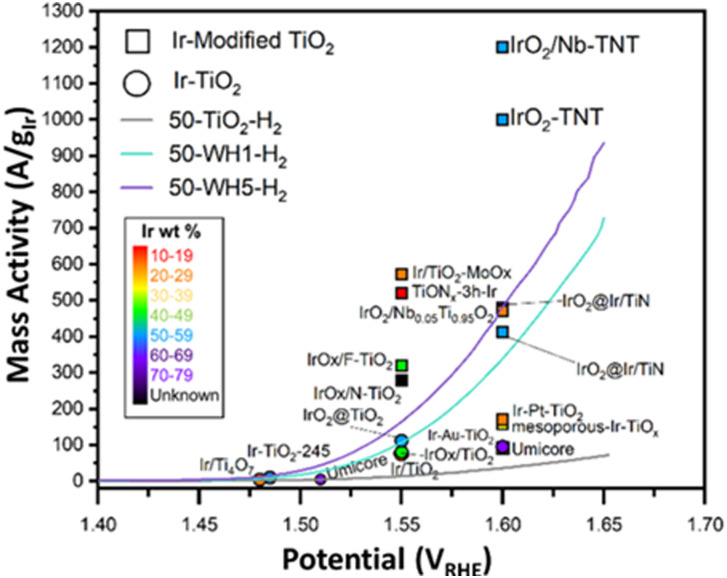
Mass activity of various Ir–TiO_2_ catalysts^9,12,22,26,28,36,54–60^ for the OER in acidic electrolyte compared to the results for the materials in this work (50-TiO_2_-H_2_, 50-WH1-H_2_, 50-WH5-H_2_). The circular symbols indicate Ir–TiO_2_ catalysts only, whereas the square symbols represent Ir-modified TiO_2_ (*e.g.*, the addition of either metal or non-metal elements to the TiO_2_ support). The different colours detail the Ir wt%.

To assess Ir utilisation, mass activities (A g_Ir_^−1^) were calculated (Fig. 1B) using the theoretical Ir loading (details provided in the ESI†). Overall, catalysts with 50 wt% Ir have much higher mass activities than 25 wt%. For example, 50-WH5-H_2_ has a mass activity of 936 A g_Ir_^−1^ compared to 25-WH5-H_2_ with 49 A g_Ir_^−1^ at 1.65 V_RHE_. To evaluate the role of AuPd loading as a conductive additive to the support structure, two different AuPd loadings (WH1 and WH5) with 50 wt% Ir were compared at 1.65 V_RHE_. We observed that 50-WH5-H_2_ achieved a higher mass activity (936 A g_Ir_^−1^) than 50-WH1-H_2_ (728 A g_Ir_^−1^). By comparing these mass activities to 50-TiO_2_-H_2_ (72 A g_Ir_^−1^ at 1.65 V_RHE_) we highlight that the AuPd significantly enhanced the electrochemical performance of our catalysts. When the prepared supported catalysts are benchmarked against unsupported commercial IrO_2_, we observed higher mass activities for 50-WH1-H_2_ and 50-WH5-H_2_ compared to commercial IrO_2_ (Fig. 1B). Furthermore, our highest performing OER catalysts demonstrated comparable mass activities to previously reported Ir-based catalysts prepared on TiO_2_-based supports when assessed in an RDE (Fig. 2 and Table S2, Fig. S2, ESI†).

To provide a preliminary screening of catalyst stability, RDE measurements were conducted. Firstly, the supported catalysts were conditioned by cycling between 0.025–1.0 V_RHE_ at 200 mV s^−1^ for 50 cycles and subsequently by performing 10 CVs between 1.0–1.8 V_RHE_ at 10 mV s^−1^ (Fig. S3, ESI†). For the thermally reduced supported catalysts with 25 and 50 wt% Ir, we observed an increase in activity during the first 10 CVs, irrespective of the AuPd loading (Fig. S3a, ESI†). It is interesting to highlight that the thermally oxidised supported catalyst 50-WH5-air showed minimal changes in activity during the first 10 CVs (Fig. S3b, ESI†). Conversely, thermally oxidised supported catalysts 50-WH1-air and 50-TiO_2_-air showed a deactivation of catalyst activity after 10 CV cycles. Similar dynamic behaviour of activation as a function of CV cycling has been observed in previously reported Ir-based OER catalysts, and has been attributed to the electrochemical growth of a hydrous Ir which can form during potential cycling.^61,62^ To assess the kinetics of the synthesised catalyst-support, Tafel analysis was performed (Fig. S4, ESI†). For each of the catalysts prepared, two distinct Tafel regions were observed corresponding to two distinct regions of the CV: low (<300 mV) and high overpotentials (>300 mV). This has previously been attributed to the presence of different active sites, or changes to the catalyst surface (reorganisation) at different applied potentials.^9,63^

### 3.2. Conductivity measurements

**Fig. 3:**
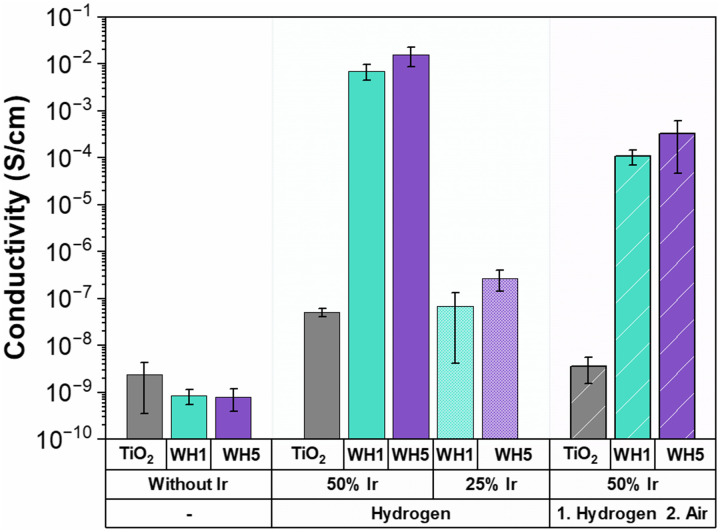
Conductivity of the various supports and catalyst-support motifs synthesised. Conductivity was measured with an in-house developed conductivity cell and are reported as an average of triplicate measurements.

Triple phase boundaries (TPBs) are regions where the electrolyte, gas and catalyst are in contact leading to the OER to occur.^64,65^ These TPBs can enhance OER performance when there is high conductivity.^65^ To probe the role of conductivity on electrochemical OER activity, conductivity measurements of the synthesised supports and supported catalysts were conducted using an in-house developed cell (Fig. 3). It is important to note that owing to the nature of the conductivity setup, these values are not absolute conductivity values, but rather a method to enable in-house comparisons. All hollow TiO_2_ supports were found to have low conductivities irrespective of the AuPd loading or thermal annealing conditions (10^−8^–10^−9^ S cm^−1^). Interestingly, when Ir is deposited onto the catalyst-supports in combination with AuPd, a higher conductivity is observed for both Ir loadings (25 wt% and 50 wt%) and under both thermal treatments (reducing or oxidised) when compared to sibling samples prepared identically, but without AuPd. The presence of Au has previously been identified as a factor in increasing conductivity as the Au thickness increases.^66,67^ Similarly, in this work the catalysts with Ir deposited on WH5 have higher conductivities (10^−2^–10^−7^ S cm^−1^) compared to those supported on WH1 (10^−3^–10^−8^ S cm^−1^). Finally, when all other synthetic parameters remain constant, we observe significantly higher conductivity (10^−2^–10^−8^) for thermally reduced samples compared to the thermally oxidised supported catalysts (10^−4^–10^−9^ S cm^−1^).

When analysing the OER performance (Fig. 1), it is noticeable that the two most conductive supported catalyst are indeed the two most active catalysts, suggesting that conductivity is a critical parameter for designing active OER catalysts. However, while the 50-WH1-air and 50-WH5-air samples have significantly higher conductivities compared to 25-WH1-H_2_ and 25-WH5-H_2_, their OER performance is rather similar. These results combined with the measured ohmic resistance (Fig. S14, ESI†) highlights the importance of obtaining optimal material composition with high conductivity to increase TPBs conditions leading to high OER performance. Thus, we can conclude that while reasonably high catalyst conductivity is essential for OER activity, such *ex situ* powder conductivity measurements are not the sole predictor for OER activity trends in these supported catalysts.

### 3.3. Catalyst-support materials characterisation

**Fig. 4:**
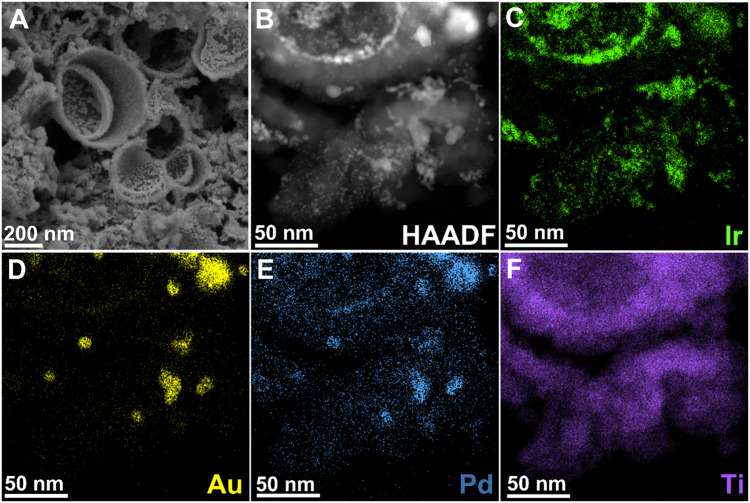
(A) SEM image of 50-WH5-H_2_, (B) HAADF-STEM image of 50-WH5-H_2_. (C)–(F) STEM-EDS elemental maps for Ir, Au, Pd and Ti, respectively.

The hollow TiO_2_ supports are observed to have an open nanoshell morphology and are ∼270 nm in diameter (Fig. 4A and Table S3, ESI†). High-angle annular dark field scanning transmission electron microscopy (HAADF-STEM) imaging coupled with energy dispersive X-ray spectroscopy (EDS) was utilised to probe the morphology (Fig. 4B) and dispersion of Ir, Au, Pd and Ti (Fig. 4C–F and Fig. S6, S9–S11, ESI†). This confirmed the presence of spherical TiO_2_ motifs, and AuPd alloy nanoparticles non-uniformly distributed across TiO_2_ for the as synthesised AuPd–TiO_2_ supports (WH1 and WH5). It is notable that the Ir, Au, and Pd are distributed both within the hollow TiO_2_ (concave surface) as well as externally (convex surface). Analysis of lattice fringes visible in high resolution transmission electron microscopy (HRTEM) images of the AuPd nanoparticles on the WH1 support (Fig. S6e, ESI†), indicates a *d*-spacing of 0.24 nm, which can be attributed to the expected {111} lattice spacings of face centred cubic (FCC) AuPd alloy.^68^

The average AuPd particle diameter in WH1 and WH5 is ∼8 nm (Table S3, ESI†) prior to Ir deposition (Fig. S6, ESI†). The Kolmogorov Smirnov tests of normality^69^ indicated that the size of AuPd particles in WH1 (as prepared) were not normally distributed whereas particles in WH5 showed normal distribution (Fig. S7, ESI†). Additional statistical analysis (chi-squared test) was used to identify the frequency of the particle sizes. By grouping the AuPd particles by size (<5, ≥5–<9, ≥9–<13, 13+), we observed a significant discrepancy (when *p* < 0.05) in the particle sizes distributions between WH1 and WH5 (*p* = 0.01). Therefore, *post hoc* Fisher's exact tests were conducted and identified that WH1 contained a significantly higher proportion of ≥5–<9 nm particles compared to WH5 which had a higher proportion of ≤5 nm particles (*p* = 0.025). The TiO_2_ hollow sphere particle sizes were measured from SEM images and show comparable average diameters of 270 nm in WH1 and 275 nm in WH5 (Table S3 and Fig. S7b, ESI†). Chi-squared analysis on TiO_2_ particle size proportion showed no significant difference (*p* = 0.53).

The morphology of the thermally reduced 25 and 50 wt% Ir loaded catalysts and the Au, Pd, Ir and Ti distributions were determined *via* SEM, HAADF-STEM imaging and STEM-EDS mapping (Fig. 4 and Fig. S9, respectively, ESI†). Using SEM, no discernible comparison could be obtained due to the limitations of electron microscopy on the small nanoparticles (Fig. S8, ESI†). A non-uniform distribution of Ir and AuPd particles across the TiO_2_ supports is observed post annealing *via* TEM. Generally, the AuPd nanoparticles appear to be co-located with Ir, while there are also some Ir agglomerates isolated from the AuPd. Additionally, we observe that the Ir appears to have been deposited both the inside (concave surface) and outside (convex surface) of the TiO_2_ open nanoshell.

**Fig. 5:**
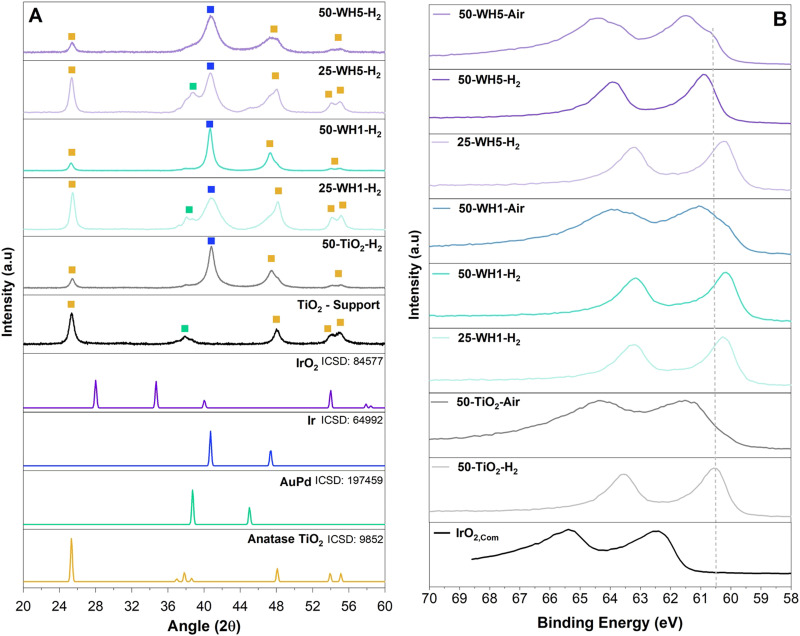
(A) XRD of the supported catalysts synthesised using 5% H_2_ in N_2_ atmosphere with reference patterns corresponding to anatase TiO_2_ (yellow), AuPd (green) and metallic Ir (blue) as shown by the symbols. The relevant ICSD collection codes are also provided. (B) XPS spectra of Ir 4f for the synthesised supported catalysts compared to a commercial IrO_2_. A dashed line is added as a guide for the reader.

The crystallinity of the supports and supported catalysts was assessed by XRD (Fig. 5A and Fig. S5, ESI†). For all samples, we observed 2*θ* peaks for crystalline anatase TiO_2_ at 25°, 48°, 54° and 55°. In all AuPd containing samples, peaks are observed at 38° and 44° corresponding to a FCC AuPd alloy. The thermally reduced samples contain a peak at 40° corresponding to FCC metallic Ir, whereas samples that were first reduced and subsequently thermally oxidised exhibited a mixture of FCC metallic Ir and low intensity rutile IrO_2_ (28° and 34.7°) (Fig. S5b, ESI†).

The crystallite size of the TiO_2_ support was assessed by the Scherrer equation and was shown to correspond approximately to the width of walls of the TiO_2_ nanospheres (∼14–16 nm). This remained constant irrespective of the composition or the synthesis environments (Table 2). Conversely, the crystallite size for Ir was found to vary across the samples. A temperature of 500 °C for H_2_ annealing was applied to the supported catalyst to ensure high crystallinity of the Ir. Crystalline Ir has been shown to improve the stability of the catalyst and adequately remove Cl^−^ impurities.^70–72^ The additional annealing in air was conducted to form a thin layer of IrO_2_ to further enhance the stability of the catalyst as metallic Ir is more unstable than IrO_2_.^73^ In both annealing environments (H_2_ and air), broader metallic Ir peaks for 50-WH5 samples indicated the presence of smaller Ir crystallites (<5 nm). We estimate that annealing in air did not significantly impact the crystallinity of Ir as we do not see the presence of IrO_2_ potentially due to amorphous IrO_2_ formed as a result of low annealing time impacting the growth of crystalline IrO_2_. Narrower Ir XRD peaks were observed for 50-WH1 and 50-TiO_2_ indicating larger Ir metal crystallites of 10–14 nm. We speculate that the lower 25 wt% Ir in both WH1 and WH5 provides sufficient anchor sites to support these smaller Ir crystalline growth. Conversely, at the higher 50 wt% Ir loading, only WH5 has sufficient anchor sites to maintain the smaller crystallite size.

**Table 2:** Crystallite sizes (calculated by Scherrer analysis) of Ir metal and TiO_2_ for the supported catalysts

Samples	Crystallite size (nm)	
	Ir	TiO_2_
25-WH1-H_2_	4	16
25-WH5-H_2_	5	15
50-TiO_2_-H_2_	10	14
50-TiO_2_-Air	12	16
50-WH1-H_2_	13	16
50-WH1-Air	14	16
50-WH5-H_2_	5	14
50-WH5-Air	5	14

XPS analysis was performed on the as-synthesised supported catalysts and a commercial rutile IrO_2_ catalyst to probe the Ir oxidation state (Fig. 5B and Fig. S12, ESI†). For all of the synthesised samples, the observed Ir 4f doublets are shifted to lower binding energies compared to a typical rutile IrO_2_.^74^ Consistent with the XRD findings, the Ir in the thermally reduced samples is more metallic in nature (lower binding energies) compared to thermally oxidised samples. Furthermore, the thermally oxidised (air) samples show the clear presence of at least two doublets corresponding to a more oxidised (*e.g.*, 4+) as well as reduced (metallic) Ir. Interestingly, no significant shift is observed in the Ir 4f region as a function of AuPd loading. The Au 4f region was also analysed. Typically, metallic Au 4f_7/2_ has a binding energy of 84.0 eV.^75^ A slight shift towards lower binding energies was observed for AuPd loaded supports (83.4 eV and 83.2 eV for WH1 and WH5, respectively) (Fig. S12a, ESI†). Upon addition of 25 wt% Ir (25-WH5-H_2_ and 25-WH1-air), the Au 4f region remains largely unchanged. However, with a higher Ir loading (50-WH5-H_2_ and 50-WH5-Air) a shift to lower Au binding energy <83.0 eV is observed, with 50-WH5 being slightly more oxidised compared to 50-WH1. Across all samples, the Ti 2p region remains unchanged, irrespective of AuPd, Ir loading and annealing environment (Fig. S12b, ESI†).^76^

### 3.4. Membrane electrode assembly performance

**Fig. 6:**
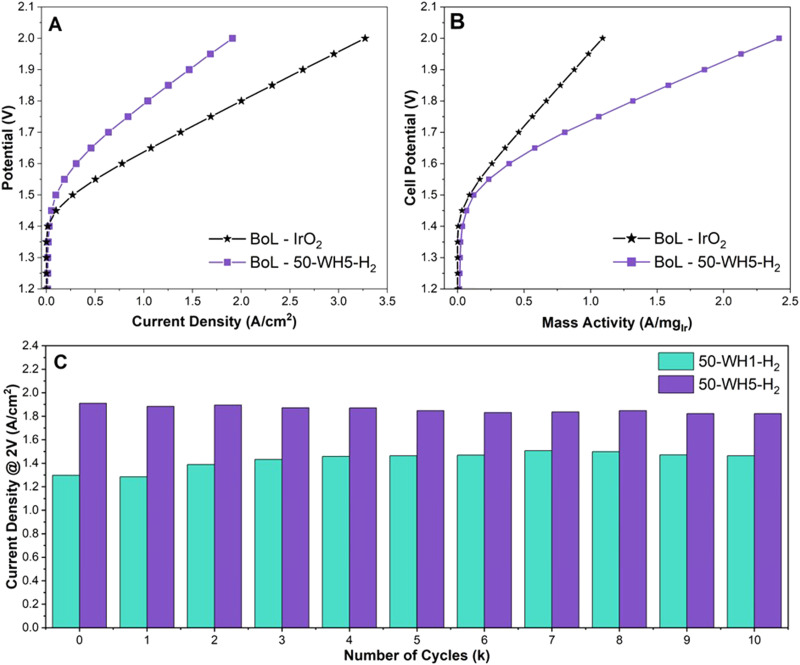
(A) Polarisation curve of proton exchange membrane water electrolyser deploying 50-WH5-H_2_ anode catalysts alongside a commercial rutile IrO_2_, (B) mass activity of the same catalysts and (C) performance of supported catalysts at 2 V after each thousandth cycle. Cell parameters: N212 membrane, 60 °C and N_2_ gas flow. OER catalyst loadings: 3 mg_IrO2_ cm^−2^ for IrO_2_ and 0.79 mg_Ir_ cm^−2^ for 50-WH5-H_2_.

To understand the relevance of our catalyst design and RDE OER performance for PEM-ELs, we integrated our highest performing catalysts (50-WH1-H_2_ and 50-WH5-H_2_) into MEAs. The samples were benchmarked against an unsupported commercial IrO_2_ (Fig. 6). Nafion 212 membrane and a commercial 50 wt% platinum on carbon (Pt/C) cathode catalysts were used throughout. The anode catalyst loadings were 1.62 mg_cat_ cm^−2^ for 50-WH1-H_2_ and 1.58 mg_cat_ cm^−2^ for 50-WH5-H_2_. The unsupported IrO_2_ had a loading of 3 mg_IrO2_ cm^−2^. Fig. 6A and Fig. S11 (ESI†) shows the beginning of life (BoL) polarization curve for prior to AST potential cycling for the supported and unsupported catalysts. To reach 1 A cm^−2^, 1.79 V is required at the BoL for 50-WH5-H_2_ whereas 50-WH1-H_2_ requires 1.91 V. The commercial IrO_2_ only required 1.63 V to reach 1 A cm^−2^. The Ir utilisation was further evaluated by calculating Ir-mass activity (Fig. 6B and Fig. S11b, ESI†). We observe that both supported catalysts achieved superior mass activity compared to unsupported commercial IrO_2_ at 2 V (1.09 A mg_IrO2_^−1^). Specifically, 50-WH5-H_2_ achieved higher mass activities (2.42 A mg_Ir_^−1^ at 2 V) compared to 50-WH1-H_2_ (1.60 A mg_Ir_^−1^ at 2 V), indicating the critical role of the higher AuPd loading for OER activity. We also speculate that the smaller crystallite size present in 50-WH5-H_2_ leads to a higher performance MEA owing to a larger Ir surface area. More critically, we highlight that these mass activity trends in MEA agree with RDE mass activity measurements (Fig. 1 and Table S4, ESI†) where both supported catalysts exhibited higher mass activity than commercial IrO_2_.

To assess catalyst stability in the single-cell MEA, we performed saw-tooth voltage cycling at 50 mV s^−1^ from 1.2–2 V for 10 000 cycles (Fig. 6C and Fig. S13, ESI†). The current density to reach 2 V was analysed for both 50-WH1-H_2_ and 50-WH5-H_2_. 50-WH5-H_2_ showed a BoL current density of 1.91 A cm^−2^ and 1.82 A cm^−2^ after 10 000 (10k) cycles (end of life, EoL). Conversely, 50-WH1-H_2_ at 2 V showed a BoL of 1.30 A cm^−2^ and EoL of 1.46 A cm^−2^ at 2 V. Interestingly, 50-WH1-H_2_ increased in performance at potentials >1.8 V after 10k cycles. Studies have shown that dissolution and redeposition of catalysts leads to improved OER performance due to the presence of a self-assembled catalyst layer.^8–10^ This could be due to either increase in Ir surface area or improved mass transport due to catalyst redeposition within the catalyst layer during AST potential cycling. Conversely, 50-WH5-H_2_ shows increased potential across current densities post AST. However, a voltage decay of 3 μV per cycle at 1 A cm^−2^ indicates minimal performance loss during the AST, suggesting that this catalyst is highly stable even in a MEA configuration. Direct comparison of MEA decay rates from literature are complicated by the differences in cell setup and AST profiles. The desired degradation rate for potential GW scale application of electrolysers is <6 μV h^−1^ and our results indicate that these TiO_2_ supported Ir catalysts exceed the desired degradation rates.^39^ However, chronopotentiometric holds exceeding thousands of hours at >1 A cm^−2^ instead of potential sweeps utilised here are necessary to empirically demonstrate such durabilities. Such long-term durabilities are deemed not viable in academic research laboratories and are better suited for commercial feasibility studies in the future.

The higher AuPd content in the support clearly has a positive influence on the performance and durability of the Ir catalysts when assessed in both MEA and RDE configurations. When benchmarked against previously reported supported Ir catalysts tested in an MEA (Table S4, ESI†), we observed that our catalysts required lower potentials to reach 1 A cm^−2^ compared to majority of the other catalysts, some with even higher anode catalyst loadings (>2 mg_Ir_ cm^−2^). However, we note that variations in cell temperature, membrane and testing conditions have a significant impact on the performance and thus a direct comparison (as we have done for RDE testing) is not possible.

To evaluate Ir dissolution in the MEAs, a sample of the test water was collected at the end of the AST and analysed using ICP-MS. We observed that Ir dissolution was highest for better performing 50-WH5-H_2_ (3.6 μg L^−1^) compared to 50-WH1-H_2_ (1.8 μg L^−1^) after 10k cycles. The increased Ir dissolution for 50-WH5-H_2_ may, at least to some extent, account for the decrease in activity during the AST 10k cycles (Fig. 6C). We hypothesise that the larger Ir crystallite size (∼13 nm) in 50-WH1-H_2_ could leach at slower rate than the smaller crystallites (∼5 nm) in 50-WH5-H_2_. It is also plausible that the larger Ir particles in 50-WH1-H_2_ could be undergoing nanostructuring leading to higher surface area under the prevailing electrochemical conditions, and thus may explain the observed improved performance post-AST. We note that Ir is potentially redeposited onto the support rather than irreversible leaching.^77^ It has been shown previously that most of the leached Ir is likely trapped within the membrane, transport layers and catalyst layers rather than entering the water feed, meaning that the majority of the trapped Ir will not be detected in our ICP analysis.^78^

To assess any changes in the oxidation state during the AST, we conducted EoL XPS analysis of the tested MEAs (post AST) which were compared to the as synthesised catalyst powders (Fig. S14, ESI†). In both supported catalysts, the Ti 2p region remains unchanged post AST when compared to the as synthesised catalyst powder (Fig. S14c, ESI†). As discussed previously, the Ir surface of the as synthesised 50-WH1-H_2_ and 50-WH5-H_2_ was predominantly metallic in nature (Fig. 5B). However, a slightly lower binding energy was observed for 50-WH1-H_2_ (∼60.2 eV) compared to 50-WH5-H_2_ (∼60.8 eV). Post AST, 50-WH1-H_2_ was shown to have shifted slightly to higher binding energy (∼0.2 eV) indicating a more oxidised surface. Conversely, 50-WH5-H_2_ Ir 4f peaks were shifted to lower binding energy (∼0.7 eV) suggesting a more reduced catalyst surface. Interestingly, the Au 4f_7/2_ (Fig. S14b, ESI†) region showed lower binding energies (more metallic) for 50-WH1-H_2_ compared to 50-WH5-H_2_ in the as prepared powders as well as post AST. For both samples, the peaks shift to a higher binding energy post AST indicating that the Au is oxidised.

## 4. Conclusion

We demonstrate a unique, hollow TiO_2_ structure decorated with AuPd nanoparticles as a high-performance catalyst-support for iridium-based OER electrocatalysts. We show that the AuPd, Ir loadings and thermal annealing environment are critical to OER performance. Furthermore, catalysts prepared with a higher AuPd and Ir loading outperform those prepared with lower loadings. Specifically, the highest performing catalyst-support exhibited 3-times higher mass activity at 1.65 V_RHE_ (936 A g_Ir_^−1^) compared to commercial IrO_2_ (271 A g_Ir_^−1^) in half-cell RDE testing. The highest performing catalysts were integrated into a membrane electrode assembly and subjected to 10k accelerated stress test consisting of saw-tooth potential sweep cycles. The same trend in OER activity were found to translate from the half-cell measurements to full devices (50-WH5-H_2_ > 50-WH1-H_2_). Our highest performing catalyst (50-WH5-H_2_) by RDE testing showed the highest mass activity in an electrolyser achieving 2.42 A mg_Ir_^−1^ at cell potential of 2 V compared to 1.09 A mg_IrO2_^−1^ for commercial IrO_2_. Even more impressively, our catalyst showed state-of-the-art device durabilities when compared to reported values under analogous electrolyser architecture, operating conditions and accelerated stress test protocols. Future studies will further investigate this class of OER catalysts through additional durability testing (chronopotentiometry and chronoamperometry in single cell and stack). Furthermore, intermediate compositions (between 25% and 50% Ir loading and higher Au/Pd content catalysts) will be prepared and investigated.

## Data availability

The data supporting this article will be included as part of the ESI.†

## Conflicts of interest

There are no conflicts to declare.

## Supplementary Material

EY-002-D4EY00026A-s001
