# Supplementary material for: Active and highly durable supported catalysts for proton exchange membrane electrolysers
Source: EES Catal. 2024 Jun 21;2(5):1139–51. doi: 10.1039/d4ey00026a (PMC11375952; doi:10.1039/d4ey00026a)
Supplement: EY-002-D4EY00026A-s001 [file EY-002-D4EY00026A-s001.pdf]

Electronic Supplementary Material (ESI) for EES Catalysis.
This journal is © The Royal Society of Chemistry 2024

Supporting Information

Active and highly durable supported catalysts for proton exchange membrane electrolysers

Debora Belami[1], Matthew Lindley[2], Umesh S. Jonnalagadda[3], Annie Mae Goncalves Bullock[1], Qianwenhao Fan[4], Wen Liu[4], Sarah J. Haigh[2], James Kwan[3], Yagya N. Regmi[1], Laurie A. King[1]

Faculty of Science and Engineering, Manchester Metropolitan University, Chester Street, M1 5GD, United Kingdom[1]
Department of Materials, University of Manchester, Oxford Road, Manchester, M13 9PL, United Kingdom [2]
Department of Engineering Science, University of Oxford, Parks Road, Oxford OX1 3PJ, United Kingdom [3]
School of Chemistry, Chemical Engineering and Biotechnology, Nanyang Technological University, 62 Nanyang Drive, 637459, Singapore [4]

1. Determination of powder conductivity

The electrical conductivity was calculated using Equation. S1[1] where σ is the electrical conductivity, l is the thickness of the PTFE (and the powder), A is the geometric area of the copper electrode and R is the electrical resistance.

$$\sigma = \frac{l}{AR}$$ **Equation. S1**

2. Conversion of measured potentials to reversible hydrogen electrode

The measured potentials vs. reference during electrochemical testing are converted to the reversible hydrogen electrode (RHE) according to Nernst equation (Equation. S2).

$$E_{RHE} = E_{WE} + E_{REF} + 0.059\,pH$$ **Equation. S2**

Where E_{RHE} is potential vs. RHE, E_{WE} is the measured potential of the working electrode (WE) against $Hg/HgSO_4$ reference and E_{REF} is the standard potential of the $Hg/HgSO_4$ reference electrode (0.642 V).

3. Calculation of mass specific activity

The current can be normalised to the theoretical mass of Ir in each synthesised powder to obtain Ir-mass activity (I_m, A/g_{Ir}). For the commercial standard, IrO_2, the mass activity was calculated based on the known mass of Ir within the IrO_2 rutile structure. Utilising the area of the working electrode (0.196 cm^2) and the mass of Ir, the Ir-mass activity was calculated as shown below if current densities (I_d) are known. The theoretical iridium loading onto the RDE was 5 $x10^{-6}$ g_{Ir} for all electrochemical experiments.

$$I = \frac{I_d\left(\frac{mA}{cm^2}\right) \times 0.196\ (cm^2)}{1000}\ \text{(A)}$$ **Equation. S3**

$$I_m = \frac{I\ (A)}{Mass\ of\ Ir\ (g)}\ \text{(A/g)}$$ **Equation. S4**

Table S1: Mass of gold and palladium deposited on TiO_2 supports determined by ICP-OES.

Sample Name	Mass (mg)	Volume (mL)	Conc. by ICP (mg/L)		Active metal mass (mg)		Actual loading (wt%)	
			**Au**	**Pd**	**Au**	**Pd**	**Au**	**Pd**
WH-1	10.75	50	2.48	0.56	0.12	0.03	1.17	0.26
WH-5	10.36	50	10.77	2.10	0.54	0.10	5.54	1.08

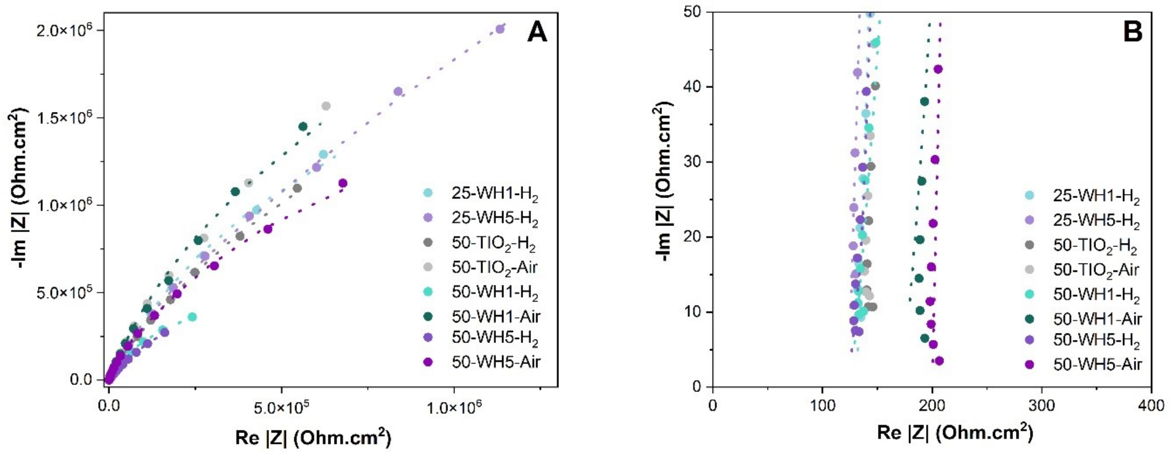

Figure S1: a) Electrochemical impedance spectroscopy (EIS) plot of supported catalysts at 100 mHz – 200 kHz from three-electrode system b) Enlarged view of the ohmic resistance from the EIS plot. Dashed lines indicate the model fit.

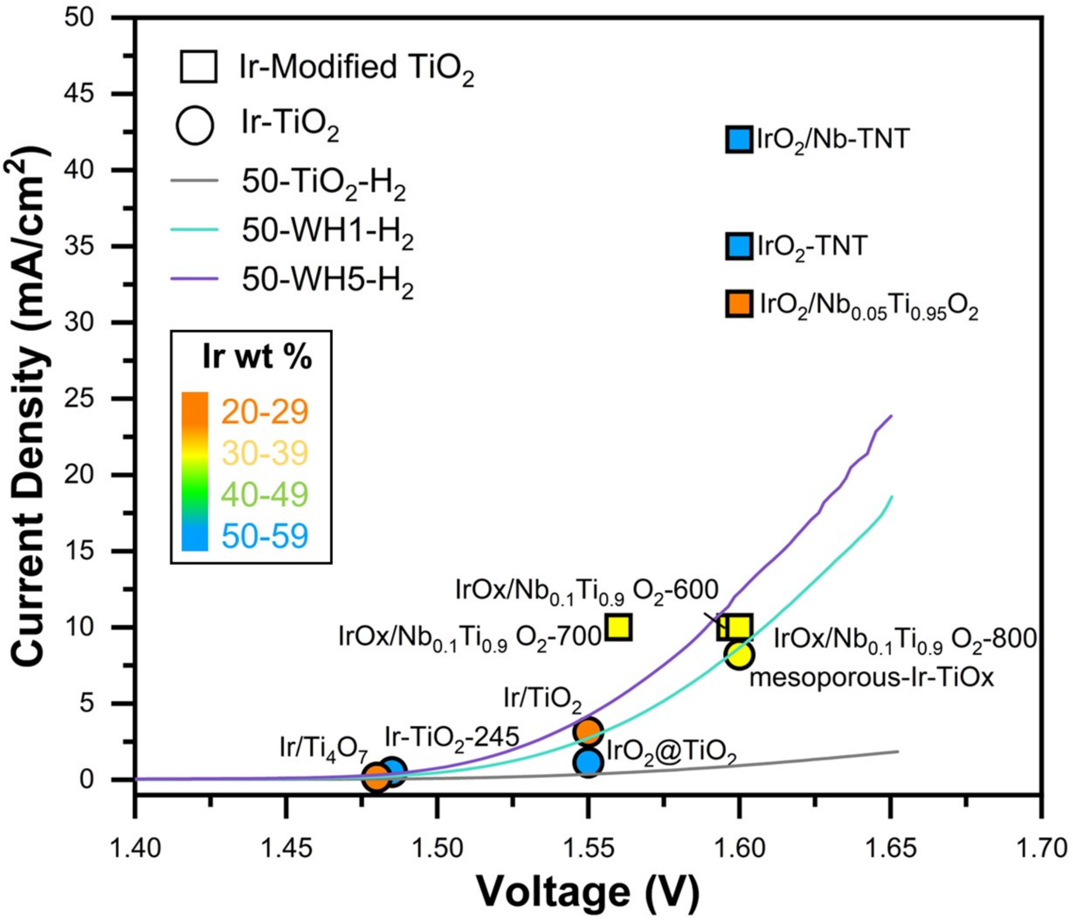

Figure S2: Current density (geometric) comparison of various literature Ir catalysts supported on or integrated into titanium containing metal oxide supports. 50-TiO_2-H_2, 50-WH1-H_2 and 50-WH5-H_2 from this work are also plotted (as lines). The literature references are shown in the supporting information as refs. 2 – 15. Symbols indicate Ir-TiO_2 catalysts only (circle) and Ir-Modified TiO_2 (square), modified relates to the addition of either metal or non-metal elements to Ti and the colour of the symbols relates to Ir wt%. The literature data is listed in further detail below in Table S2.

Table S2: Comparison of the OER activities from rotating disk electrode from the literature with mass activities and geometric area normalised current densities where available.

Sample Name	Ir wt%	Ir Loading (µg/cm²)	Electrolyte	Mass Activity (A/g_{Ir})	Current Density (mA/cm²)	Voltage at given current density (V)	Refs.
50-TiO_2-H_2	50	25.5	0.1 M $HClO_4$	72	1.84	1.65	This Work
50-WH1-H_2	50	25.5	0.1 M $HClO_4$	728	18.57	1.65	This Work
50-WH5-H_2	50	25.5	0.1 M $HClO_4$	936	23.87	1.65	This work

Ir/TiO_2-MoO_x	26	37.5	0.05 M H_2SO_4	573	-	1.55	2
Ir/TiO_2	26	37.5	0.05 M H_2SO_4	76	3.13	1.55	2
Ir-TiO_2-245	55	50	0.1 M $HClO_4$	10	0.5	1.49	3
$IrO_x/Nb_{0.1}Ti_{0.9}O_2$-600	30	-	0.5 M H_2SO_4	-	10	1.60	4
$IrO_x/Nb_{0.1}Ti_{0.9}O_2$-700	30	-	0.5 M H_2SO_4	-	10	1.56	4
$IrO_x/Nb_{0.1}Ti_{0.9}O_2$-800	30	-	0.5 M H_2SO_4	-	10	1.60	4
IrO_x/F-TiO_2	40	0.3	0.5 M H_2SO_4	320.2	-	1.55	5
IrO_x/TiO_2	40	0.3	0.5 M H_2SO_4	80.8	-	1.55	5
IrO_2-TNT	50	35	0.5 M H_2SO_4	-	35	1.60	6
IrO_2/Nb-TNT	50	35	0.5 M H_2SO_4	-	42	1.60	6
IrO_2@TiO_2	50	10	0.1 M $HClO_4$	112	1.12	1.55	7
IrO_2@Ir/TiN	50	379	0.5 M H_2SO_4	412.7	-	1.60	8
IrO_2@Ir/TiN	60	379	0.5 M H_2SO_4	480.4	-	1.60	8
Ir/Ti_4O_7	25	33.33	0.5 M H_2SO_4	4.2	0.14	1.48	9
Mesoporous-Ir-TiO_x	30	-	0.5 M H_2SO_4	158.3	8.2	1.60	10
Ir-Pt-TiO_2	25	3.49	0.1 M $HClO_4$	170	-	1.60	11
$IrO_2/Nb_{0.05}Ti_{0.95}O_2$	26	66.3	0.5 M H_2SO_4	471	31.23	1.60	12
IrO_x/N-TiO_2	-	-	0.5 M H_2SO_4	278.7	-	1.55	13
$TiON_x$-3h-Ir	11	-	0.1 M $HClO_4$	520.3	-	1.55	14
Umicore	75	3.7	0.1 M $HClO_4$	5	-	1.50	15

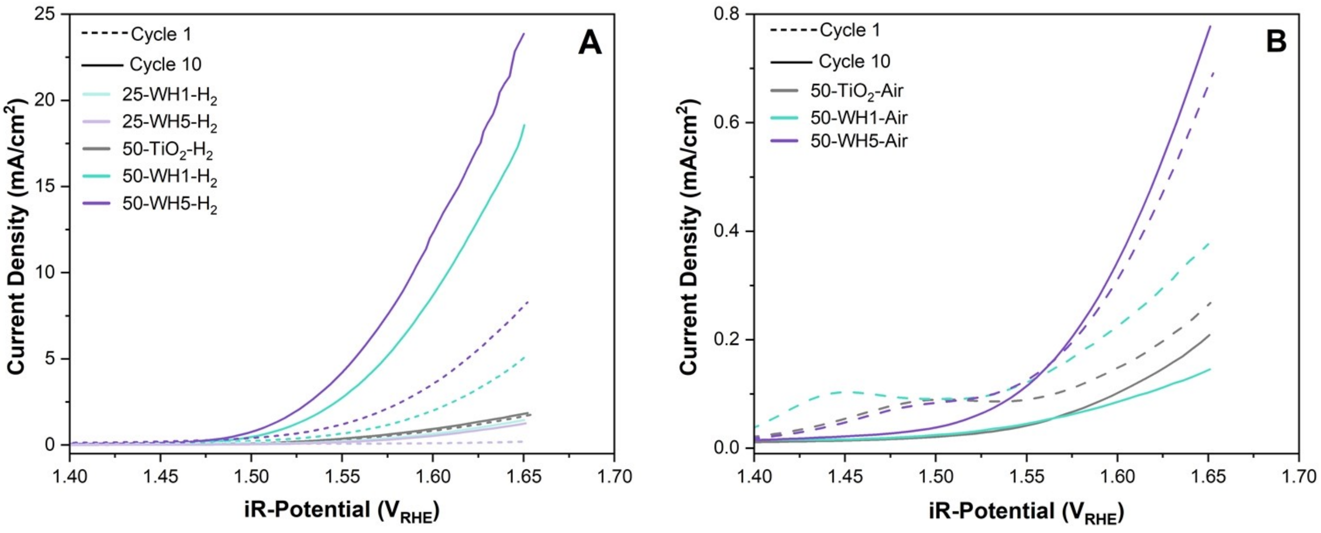

Figure S3: Stability comparison of cycle 1 and cycle 10 for supported catalysts a) thermally reduced and b) thermally oxidised. All experiments were conducted in 0.1 M $HClO_4$ electrolyte with a Au disk working electrode. Electrochemical analysis was assessed on the 1st and 10th CV cycle with theoretical Ir loading of 25.5 $\mu g_{Ir}/cm^2$. Note the differences in the magnitude of the Y-axes for A and B.

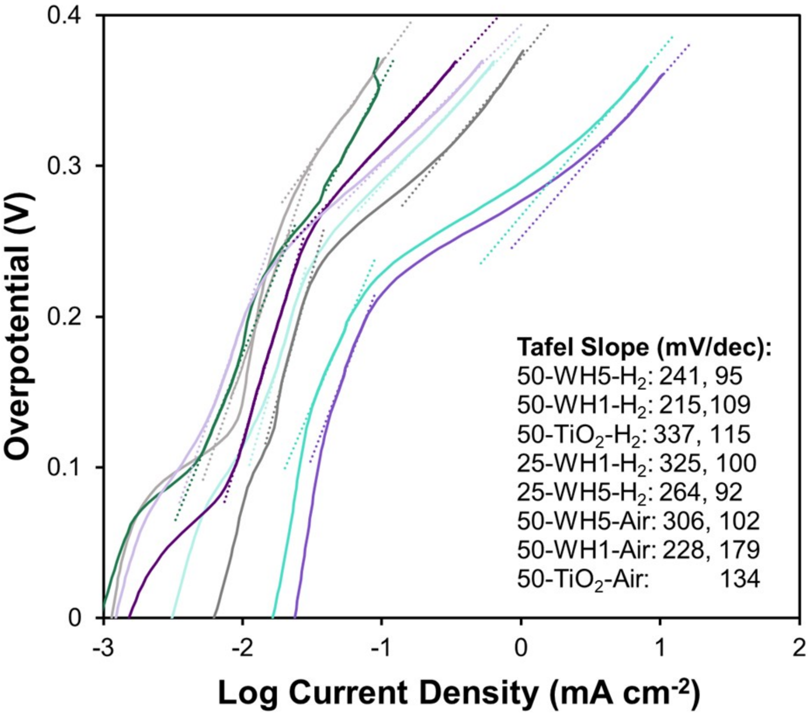

Figure S4: Tafel plots for the supported Ir catalysts with the calculated Tafel slopes.

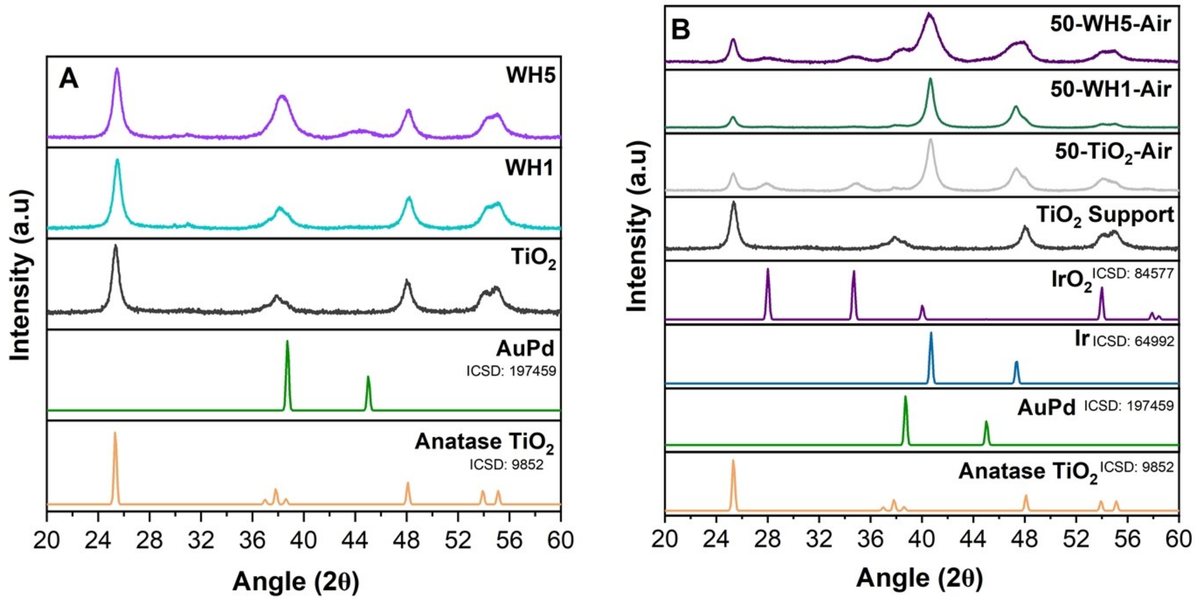

Figure S5: XRD diffraction pattern of a) supports without Ir and b) thermally oxidised TiO_2 supported Ir catalysts including reference patterns with the corresponding ICSD numbers.

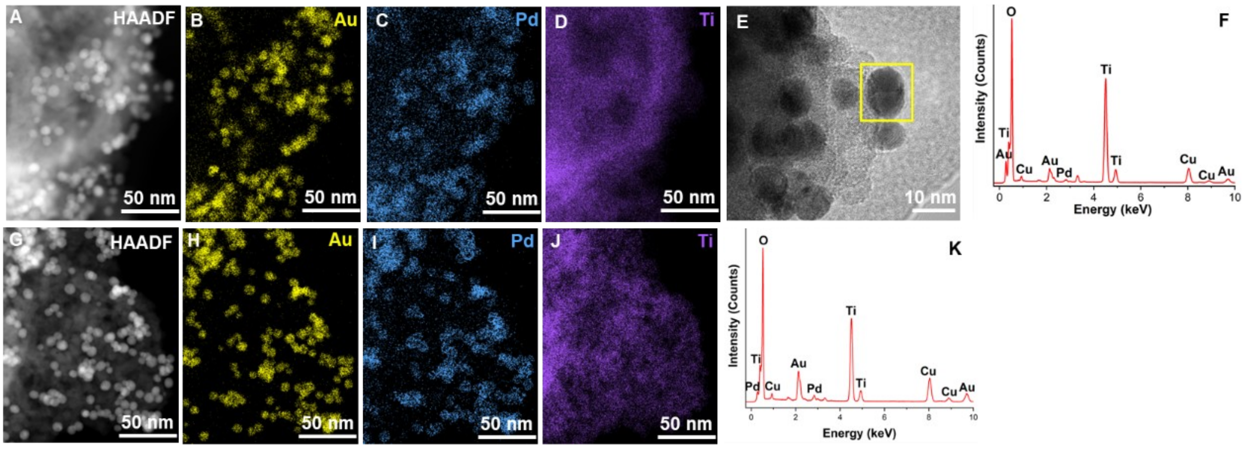

Figure S6: Characterisation of WH1 and WH5 AuPd-TiO_2 supports as synthesised (no annealing). a) HAADF-STEM image, b-d) STEM-EDX elemental maps and e) High resolution TEM image and f) summed EDS spectra for the region shown for WH1. g) HAADF STEM image, h-j) STEM-EDX elemental maps and k) summed STEM-EDX spectra for the region shown for WH5. Yellow box in (e) highlights the lattice fringe analysis region with a measured lattice spacing of 0.2 nm corresponding to the {111} spacing of AuPd alloys.

Table S3: Average (mean ± SD) AuPd nanoparticle and TiO_2 porous sphere diameters in the WH1 and WH5 catalyst-supports (prior to Ir deposition) determined from TEM and SEM.

Sample	Average AuPd particle diameter (by TEM, nm)	Average TiO_2 particle diameter (by SEM, nm)
WH1	7.5 ± 4.2	269.7 ± 55.0
WH5	8.3 ± 5.1	275.3 ± 93.0

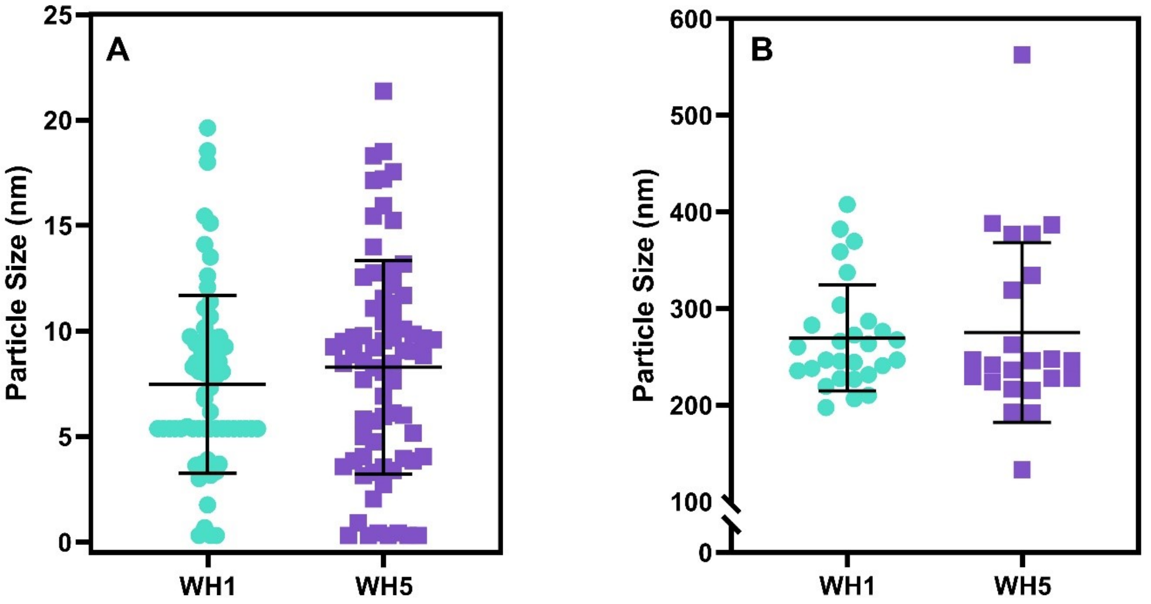

Figure S7: Particle diameter distribution obtained from (a) 67 and 72 measurements of AuPd nanoparticle size and (b) 28 and 23 measurements of the porous TiO_2 supports in WH1 and WH5, respectively. A Mann Whitney U test was used to compare the mean particle diameter between WH1 and WH5 in a) AuPd and b) TiO_2 supports. There was no significant difference between WH1 and WH5 particle size means for AuPd ($p = 0.27$) or for TiO_2 ($p = 0.59$).

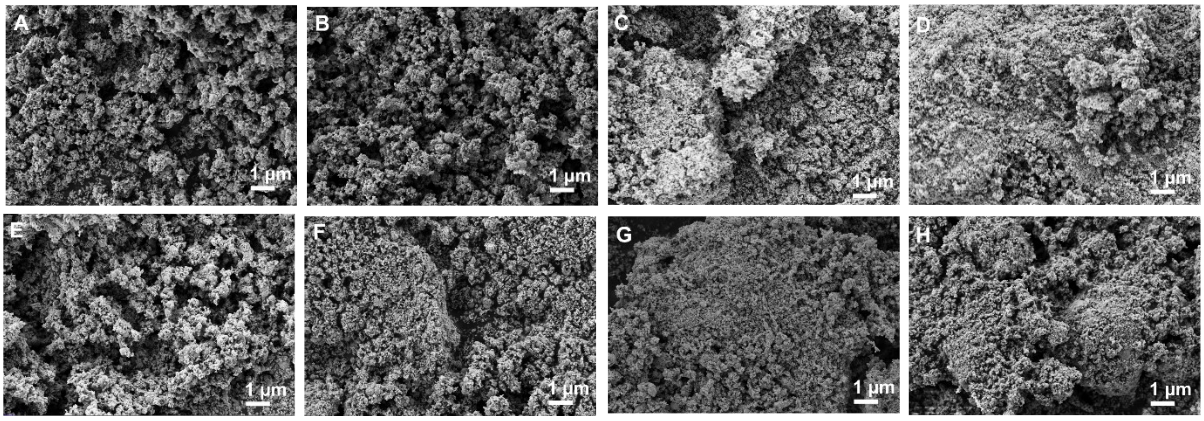

Figure S8: Scanning electron microscopy image of supported catalysts a) 25-WH1-H_2, b) 25-WH5-H_2 c) 50-TiO_2-H_2 d) 50-TiO_2-Air e) 50-WH1-H_2, f) 50-WH1-Air g) 50-WH5-H_2 and h) 50-WH5-Air

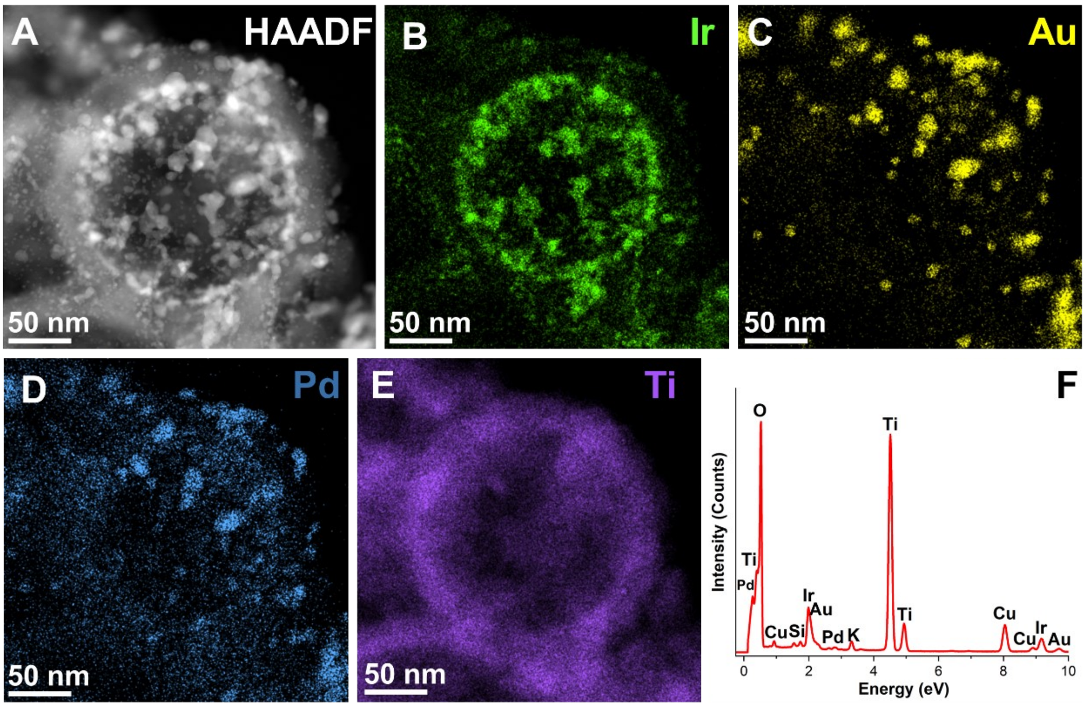

Figure S9: 25-Ir-WH5-H_2 a) HAADF-STEM image, b-e) STEM-EDX elemental maps of Ir, Au, Pd and Ti and f) Summed STEM-EDX spectra for the region shown.

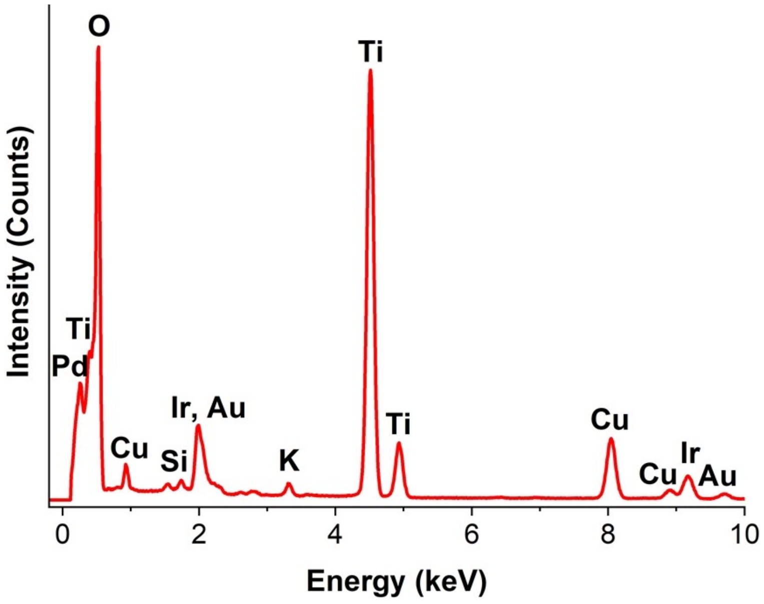

Figure S10: Summed STEM-EDX spectra for 50-WH5-H_2

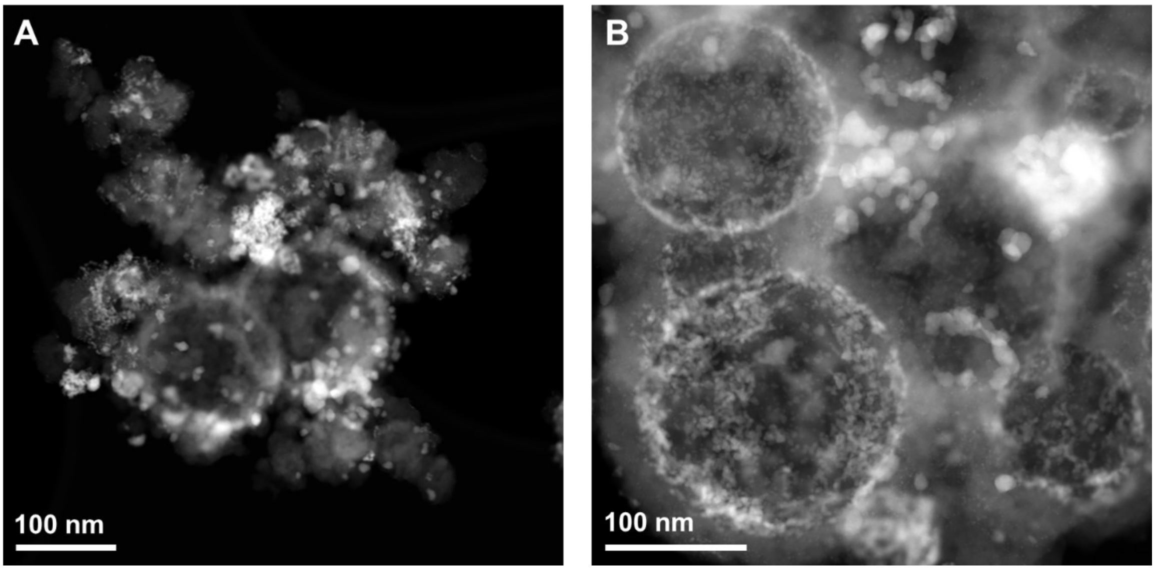

Figure S11: Further HAADF-STEM images of 50-WH5-H_2 to show other regions across the TEM grid.

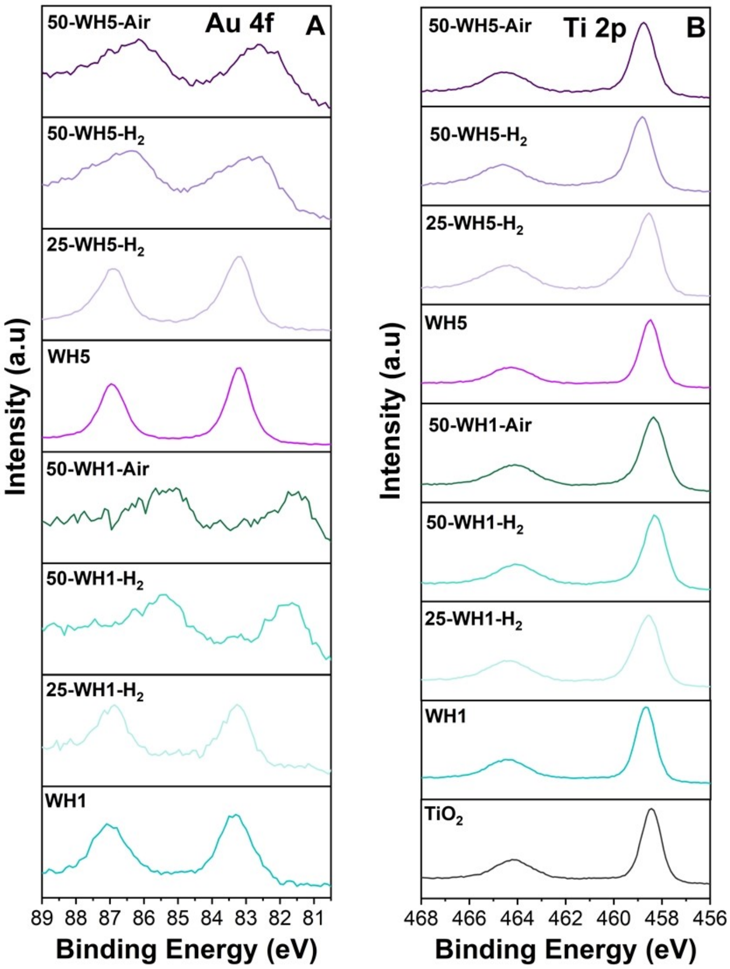

Figure S12: XPS spectra of the synthesised supported catalysts for a) Au 4f and b) Ti 2p regions.

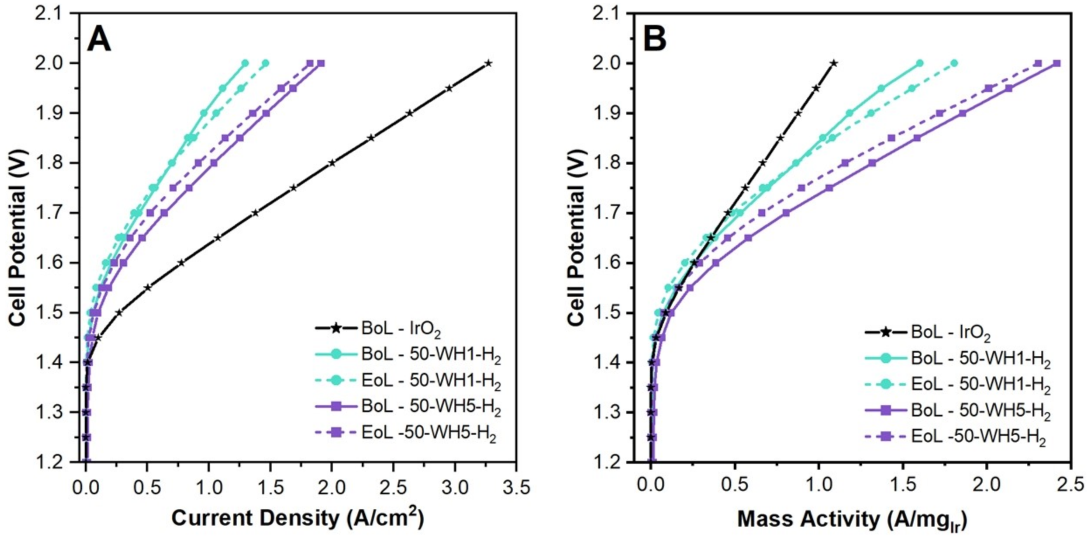

Figure S13: a) Polarisation curve of proton exchange membrane water electrolyser deploying 50-WH1-H_2 and 50-WH5-H_2 anode catalysts, b) Mass activity of unsupported and supported Ir catalysts. BoL is the beginning of life, EoL is the end of life (i.e., post 10 000 AST cycles of testing). Cell parameters: N212 membrane, 60 ^oC and N_2 gas flow. OER catalyst loadings: 3 mg_{IrO2}/cm^2 for IrO_2, 0.81 mg_{Ir}/cm^2 for 50-WH1-H_2 and 0.79 mg_{Ir}/cm^2 for 50-WH5-H_2.

Table S4: 50-WH1-H_2 and 50-WH5-H_2 accelerated stress test conditions presented alongside various literature Ir-TiO_2 based electrocatalysts. CP is chronopotentiometry and CA is chronoamperometry.

Materials	Temperature (^oC)	Membrane	Anode Catalyst Loading (mg_{Ir}/cm^2)	Potential at 1 A/cm^2		Stability Test Conditions	Refs.
				Beginning of Life (V)	End of Life (V)		
50-WH1-H_2	60	212	0.81	1.91	1.88	Saw-tooth	This Work
50-WH5-H_2	60	212	0.79	1.79	1.82	Saw-tooth	This Work
Ir/TiO_2-MoO_x	80	115	0.50	1.74	1.85	Hold	[2]
IrO_x/F-TiO_2	80	115	1.00	1.66	1.68	Hold	[5]
Ir-Pt-TiO_2-PC-ann	80	117	0.25	1.87	-	-	[11]
IrO_2@TiO_2	80	212	1.20	1.67	1.85	Hold	[16]
40I/TN-20	80	117	2.50	2.03	-	-	[17]
40Ir/V doped Ti	80	117	2.50	2.03	2.13	Saw-tooth	[18]
IrO_2/TNO-H750	80	117	2.50	1.83	1.83	Hold	[19]
40IrO_2/$Ti_{0.7}Ta_{0.3}O_2$	80	117	1.50	1.95	-	-	[20]
TiO_2-R200M	120	117	0.90	1.67	-	-	[21]
TiO_2-P25	120	117	0.90	1.75	-	-	[21]
F68-$Ir_{0.6}Sn_{0.4}O$	80	115	0.88	1.62	-	Hold	[22]

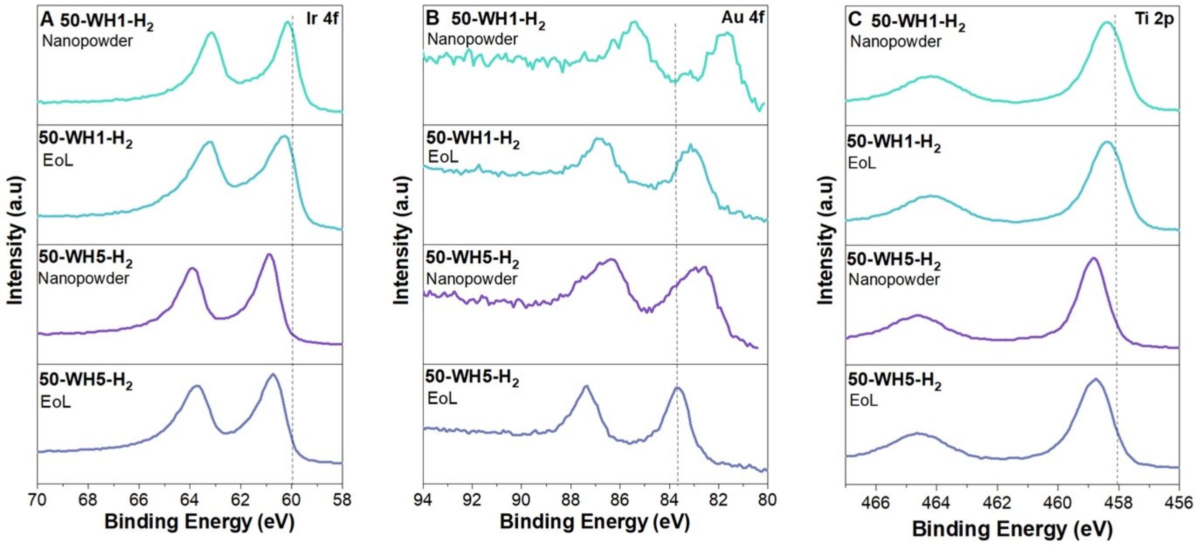

Figure S14: XPS spectra for a) Ir 4f, b) Au 4f and c) Ti 2p regions of the 50-WH1-H_2 and 50-WH5-H_2 in the form of nanopowder and post 10k AST catalyst coated membranes (EoL). Grey dashed lines are provided to guide the eye. The nanopowder spectra are taken from Figures 5b and S12.

References:

1 D. Xu, M. B. Stevens, M. R. Cosby, S. Z. Oener, A. M. Smith, L. J. Enman, K. E. Ayers, C. B. Capuano, J. N. Renner, N. Danilovic, Y. Li, H. Wang, Q. Zhang and S. W. Boettcher, Earth-Abundant Oxygen Electrocatalysts for Alkaline Anion-Exchange-Membrane Water Electrolysis: Effects of Catalyst Conductivity and Comparison with Performance in Three-Electrode Cells, *ACS Catal.*, 2019, **9**, 7–15.

2 E. J. Kim, J. Shin, J. Bak, S. J. Lee, K. hyun Kim, D. H. Song, J. H. Roh, Y. Lee, H. W. Kim, K. S. Lee and E. A. Cho, Stabilizing role of Mo in TiO2-MoOx supported Ir catalyst toward oxygen evolution reaction, *Appl. Catal. B Environ.*, 2021, **280**, 1–10.

3 E. Oakton, D. Lebedev, M. Povia, D. F. Abbott, E. Fabbri, A. Fedorov, M. Nachtegaal, C. Copéret and T. J. Schmidt, IrO2-TiO2: A High-Surface-Area, Active, and Stable Electrocatalyst for the Oxygen Evolution Reaction, *ACS Catal.*, 2017, **7**, 2346–2352.

4 J. Liao, Y. Wang, M. Chen, M. Wang, J. Fan, H. Li, H. Wang, L. Zeng and T. Zhao, IrOXSupported onto Niobium-Doped Titanium Dioxide as an Anode Reversal Tolerant Electrocatalyst for Proton Exchange Membrane Fuel Cells, *ACS Appl. Energy Mater.*, 2022, **5**, 3259–3268.

5 G. Li, X. Xu, H. Liu, X. Yang and M. C. Lin, Enhanced Electrocatalytic Performance of IrOx by Employing F-Doped TiO2 as Support towards Acidic Oxygen Evolution Reaction, *ChemCatChem*, 2022, **14**, 1–7.

6 R. V. Genova-Koleva, F. Alcaide, G. Álvarez, P. L. Cabot, H. J. Grande, M. V. Martínez-Huerta and O. Miguel, Supporting IrO2 and IrRuOx nanoparticles on TiO2 and Nb-doped TiO2 nanotubes as electrocatalysts for the oxygen evolution reaction, *J. Energy Chem.*, 2019, **34**, 227–239.

7 C. Van Pham, M. Bühler, J. Knöppel, M. Bierling, D. Seeberger, D. Escalera-López, K. J. J. Mayrhofer, S. Cherevko and S. Thiele, IrO2 coated TiO2 core-shell microparticles advance performance of low loading proton exchange membrane water electrolyzers, *Appl. Catal. B Environ.*, 2020, **269**, 118762.

8 G. Li, K. Li, L. Yang, J. Chang, R. Ma, Z. Wu, J. Ge, C. Liu and W. Xing, Boosted Performance of Ir Species by Employing TiN as the Support toward Oxygen Evolution Reaction, *ACS Appl. Mater. Interfaces*, 2018, **10**, 38117–38124.

9 T. Morawietz, R. Hiesgen, S. S. Hosseiny, A. S. Gago and K. A. Friedrich, Nanostructured Ir-supported on Ti4O7 as a cost-effective anode for proton exchange membrane (PEM) electrolyzers, 2016, 4487–4495.

10 D. Bernsmeier, M. Bernicke, R. Schmack, R. Sachse, B. Paul, A. Bergmann, P. Strasser, E. Ortel and R. Kraehnert, Oxygen Evolution Catalysts Based on Ir–Ti Mixed Oxides with Templated Mesopore Structure: Impact of Ir on Activity and Conductivity, *ChemSusChem*, 2018, **11**, 2367–2374.

11 Y. N. Regmi, E. Tzanetopoulos, G. Zeng, X. Peng, D. I. Kushner, T. A. Kistler, L. A. King and N. Danilovic, Supported Oxygen Evolution Catalysts by Design: Toward Lower Precious Metal Loading and Improved Conductivity in Proton Exchange Membrane Water Electrolyzers, *ACS Catal.*, 2020, **10**, 13125–13135.

12 W. Hu, S. Chen and Q. Xia, IrO2/Nb-TiO2 electrocatalyst for oxygen evolution reaction in acidic medium, *Int. J. Hydrogen Energy*, 2014, 6967–6976.

13 G. Li, H. Jia, H. Liu, X. Yang and M. C. Lin, Nanostructured IrOx supported on N-doped TiO2 as an efficient electrocatalyst towards acidic oxygen evolution reaction, *RSC Adv.*, 2022, **12**, 28929–28936.

14 L. Moriau, M. Bele, Ž. Marinko, F. Ruiz-Zepeda, G. Koderman Podboršek, M. Šala, A. K. Šurca, J. Kovač, I. Arčon, P. Jovanovič, N. Hodnik and L. Suhadolnik, Effect of the Morphology of the High-Surface-Area Support on the Performance of the Oxygen-Evolution Reaction for Iridium Nanoparticles, *ACS Catal.*, 2021, **11**, 670–681.

15 A. Hartig-Weiss, M. Miller, H. Beyer, A. Schmitt, A. Siebel, A. T. S. Freiberg, H. A. Gasteiger and H. A. El-Sayed, Iridium Oxide Catalyst Supported on Antimony-Doped Tin Oxide for High Oxygen Evolution Reaction Activity in Acidic Media, *ACS Appl. Nano Mater.*, 2020, **3**, 2185–2196.

16 C. Van Pham, M. Bühler, J. Knöppel, M. Bierling, D. Seeberger, D. Escalera-López, K. J. J. Mayrhofer, S. Cherevko and S. Thiele, IrO2 coated TiO2 core-shell microparticles advance performance of low loading proton exchange membrane water electrolyzers, *Appl. Catal. B Environ.*, 2020, **269**, 118762.

17 C. Hao, H. Lv, C. Mi, Y. Song and J. Ma, Investigation of Mesoporous Niobium-Doped TiO2 as an Oxygen Evolution Catalyst Support in an SPE Water Electrolyzer, *ACS Sustain. Chem. Eng.*, 2016, **4**, 746–756.

18 C. Hao, H. Lv, Q. Zhao, B. Li, C. Zhang, C. Mi, Y. Song and J. Ma, Investigation of V-doped TiO2 as an anodic catalyst support for SPE water electrolysis, *Int. J. Hydrogen Energy*, 2017, **42**, 9384–9395.

19 H. Lv, S. Wang, C. Hao, W. Zhou, J. Li, M. Xue and C. Zhang, Oxygen-Deficient Ti 0.9 Nb 0.1 O 2-x as an Efficient Anodic Catalyst Support for PEM Water Electrolyzer, *ChemCatChem*, 2019, **11**, 2511–2519.

20 H. Lv, G. Zhang, C. Hao, C. Mi, W. Zhou, D. Yang, B. Li and C. Zhang, Activity of IrO2 supported on tantalum-doped TiO2 electrocatalyst for solid polymer electrolyte water electrolyzer, *RSC Adv.*, 2017, **7**, 40427–40436.

21 P. Mazúr, J. Polonský, M. Paidar and K. Bouzek, Non-conductive TiO 2 as the anode catalyst support for PEM water electrolysis, *Int. J. Hydrogen Energy*, 2012, **37**, 12081–12088.

22 G. Li, H. Yu, D. Yang, J. Chi, X. Wang, S. Sun, Z. Shao and B. Yi, Iridium-Tin oxide solid-

solution nanocatalysts with enhanced activity and stability for oxygen evolution, *J. Power Sources*, 2016, **325**, 15–24.
